# Age-Related Electrophysiological Reorganization During In-Phase and Anti-Phase Bimanual Coordination

**DOI:** 10.3390/brainsci16101075

**Published:** 2026-10-07

**Authors:** Liqing Liu, Jiale Wu, Zhihui Zhao, Qing Liu, Xuejun Bai

**Affiliations:** 1 Faculty of Psychology, Tianjin Normal University, Tianjin 300387, China; lqliu@tjnu.edu.cn (L.L.); wujiale@stu.tjnu.edu.cn (J.W.); 20250108zzh@gmail.com (Z.Z.); 2311340166@stu.tjnu.edu.cn (Q.L.); 2 Key Research Base of Humanities and Social Sciences of the Ministry of Education, Academy of Psychology and Behavior, Tianjin Normal University, Tianjin 300387, China; 3 Tianjin Social Science Laboratory of Students’ Mental Development and Learning, Tianjin 300387, China

**Keywords:** healthy aging, bimanual coordination, electroencephalography (EEG), event-related potentials, beta oscillations, event-related desynchronization (ERD)

## Abstract

**Highlights:**

**What are the main findings?**

- The older group showed poorer discrete bimanual coordination performance, with generalized slowing and a disproportionate accuracy and efficiency cost during anti-phase coordination.
- Aging was associated with electrophysiological reorganization across multiple processing stages, including larger P1 responses, a more anterior sensor-level P3 distribution, higher prestimulus beta power, and greater cumulative beta ERD burden during demanding anti-phase coordination.

**What are the implications of the main findings?**

- Anti-phase bimanual coordination may serve as a sensitive behavioral and electrophysiological probe for detecting age-related changes in cognitive–motor control.

**Abstract:**

**Background/Objectives:** Healthy aging is associated with declines in bimanual coordination, particularly under more demanding coordination modes. However, the electrophysiological changes accompanying these behavioral differences and their relevance to performance remain unclear. This study examined age-group differences in behavioral performance, ERP responses, and beta-band dynamics during in-phase and anti-phase bimanual coordination. **Methods:** Forty-two right-handed adults were included in the primary analyses of a bimanual coordination task in both in-phase and anti-phase coordination modes. Behavioral outcomes included accuracy, reaction time, and inverse efficiency scores. EEG measures included P1, N1, and P3 responses, prestimulus beta power, fixed-window relative beta power, baseline-adjusted absolute beta power, and individualized cumulative event-related desynchronization burden. **Results:** The older group was slower and less efficient overall, with disproportionate accuracy and efficiency costs during anti-phase coordination. Electrophysiologically, aging was associated with larger early P1 responses, a sensor-level redistribution of P3 amplitude and latency, and higher prestimulus beta activity. Fixed-window relative beta and baseline-adjusted absolute beta showed age-group differences without corrected coordination effects, whereas individualized cumulative ERD burden showed age-group, coordination, and interaction effects. **Conclusions:** Healthy aging was associated with poorer discrete bimanual coordination performance, altered early visual and late positive ERP responses, and stronger cumulative beta suppression under demanding anti-phase coordination.

## 1. Introduction

Bimanual coordination is the ability to execute coordinated movements with both hands in a spatially and temporally organized manner [1]. This skill is essential for many daily activities, such as tying shoelaces, opening containers, typing on a keyboard, and manipulating objects. A key distinction in the study of bimanual coordination is the relationship between the two hands during movement execution. In-phase coordination involves symmetrical activation of corresponding muscle groups in both hands, leading to stable and efficient performance. This type of coordination is usually characterized by movements directed toward mirror-symmetric spatial positions. In contrast, anti-phase coordination entails alternating activation of the corresponding muscles, resulting in a different spatial relationship between the hands, such as parallel trajectories in a horizontal side-by-side arrangement. Anti-phase coordination requires greater demands for temporal control, interlimb integration, and cognitive monitoring [2,3]. As a result, it is typically less stable and more challenging to perform than in-phase coordination, especially as task complexity increases [3,4].

Aging is associated with poorer performance in complex bimanual tasks. Older adults commonly show slower responses, lower accuracy, and greater difficulty coordinating both hands across a range of motor tasks [5,6,7]. Age-group differences are often more pronounced in asymmetric tasks, in which successful performance depends on the interaction of motor execution with temporal coordination, response selection, attentional monitoring, and stimulus–response mapping [5,8]. Anti-phase coordination may also require suppression of the intrinsic tendency toward symmetric interlimb coupling [3]. Age-related changes in these interacting processes may contribute to the significant difficulty observed during complex bimanual coordination; this effect should not be attributed solely to executive decline.

Neuroimaging studies indicate that bimanual coordination recruits a distributed sensorimotor network that includes the primary motor cortex, premotor cortex, supplementary motor area, posterior-parietal cortex, cingulate motor area, and cerebellum [9,10,11]. Compared with younger adults, older adults often exhibit greater activation across both motor and non-motor regions during coordination tasks [12,13]. The functional significance of this age-related overactivation is debated: it may reflect compensation that supports performance [14], dedifferentiation indicative of less efficient and less specialized processing [15,16], or a resource-limited over-recruitment that becomes insufficient under high demands [17]. Determining whether such neural changes are adaptive or maladaptive under increased coordination complexity remains a key question that can be addressed by linking neural activity directly to behavioral performance.

Electrophysiological measures provide a useful approach for addressing this question because they capture neural processing at multiple stages of task performance. Event-related potentials (ERPs) can distinguish early sensory-attentional processing from later cognitive and response-selection processes. Early visual components such as P1 and N1 are sensitive to attentional allocation and perceptual discrimination, whereas the P3 component reflects a set of partially overlapping processes related to stimulus evaluation, context updating, task relevance, and stimulus–response processing [18,19]. Age-related changes in P3 amplitude, latency, and scalp distribution have frequently been interpreted as evidence of altered cognitive control and task evaluation processes [20]. In parallel, time–frequency analyses have identified beta-band oscillations as an important feature of motor preparation and execution. Task-related beta event-related desynchronization (ERD) refers to a reduction in beta power relative to a reference period and has been linked to changes in the current sensorimotor state during movement preparation, response selection, and movement execution [21,22]. Some studies have reported stronger or more spatially extensive beta ERD in older adults during bimanual movement, particularly under more demanding coordination modes [23,24]. ERD timing can nevertheless vary across individuals, and age-related beta patterns may differ across movement stages. Methodological work has therefore used participant-specific temporal features and the area under the desynchronization curve to summarize ERD dynamics [25]. This individualized integration approach provides a useful complement to a common fixed analysis window, without implying that the resulting cumulative measure is an established physiological construct. Importantly, age differences may already be present before stimulus onset, because higher spontaneous or prestimulus beta activity has been reported in older adults [26,27,28]. Prestimulus beta level should therefore be considered when interpreting task-period oscillatory effects.

Despite increasing evidence of age-related changes in both ERPs and oscillatory activity during motor tasks, there are few studies that have jointly examined early visual ERPs, late positive activity, and beta-band dynamics in younger and older adults performing the same discrete bimanual choice-reaction task. As a result, it remains unclear whether aging primarily affects early cue processing, later evaluative and response-selection processes, motor-related neural dynamics, or multiple stages of processing simultaneously. Moreover, the relationship between age-related neural recruitment and behavioral performance remains insufficiently understood, particularly under conditions of increased coordination complexity and when response speed and overall behavioral efficiency are considered separately.

The present study addressed these questions by combining behavioral measures, ERP analyses, and time–frequency analyses during in-phase and anti-phase bimanual coordination. Particular attention was given to age-related differences in prestimulus beta activity, early cue processing (P1 and N1), late evaluative processing (P3), and task-related beta desynchronization. For beta ERD, we examined both power changes within a common task window and cumulative ERD burden, defined here as the time-integrated magnitude of below-baseline beta suppression within each individually identified ERD episode. Based on previous findings, we expected older adults to show poorer behavioral performance, especially during anti-phase coordination. At the neural level, we anticipated age-related alterations in late-stage processing, reflected by a more anterior distribution of P3 activity and enhanced beta desynchronization. Finally, we examined whether variation in these neural measures was related to response speed and overall behavioral efficiency.

## 2. Materials and Methods

### 2.1. Participants

This study used a 2 × 2 mixed design, with age group as the between-participant factor and coordination mode as the within-participant factor. G*Power 3.1.9.7 [29] was used to calculate the sample size, with a significance level of 0.05 and a medium effect size (*f* = 0.25). The assumed effect size (*f* = 0.25) was based on previous ERP and behavioral studies examining age-related differences in bimanual coordination and cognitive–motor performance [12,30]. The repeated-measures correlation was set to the G*Power default of *r* = 0.50 because no task-specific estimate was available. The nonsphericity correction was set to ε = 1.00, as the within-participant factor had only two levels. The results indicated that a minimum total sample size of 34 participants (i.e., 17 per group) was necessary to achieve a statistical power level of 80%. To address the uncertainty regarding the actual statistical power, forty-five right-handed adults were recruited from Tianjin, comprising 23 younger adults (10 men; 18–26 years, *M* = 20.30 years) and 22 older adults (8 men and 14 women; 57–70 years, *M* = 64.64 years). Three older adults were excluded from the primary analyses because fewer than 30 valid EEG trials remained in the anti-phase condition after preprocessing. The final primary cohort therefore comprised 23 younger adults (10 men, 18–26 years, 20.30 ± 1.94 years) and 19 older adults (7 men, 59–70 years, 65.47 ± 3.20 years) and was used for the primary behavioral and EEG analyses. Behavioral analyses were additionally repeated in all 45 recruited participants as a supplementary robustness analysis.

All participants were right-handed, scored above 80 on the Edinburgh Handedness Scale, had normal or corrected vision, had no history of mental or neurological disorders, and scored less than 13 on the Beck Depression Scale. For older participants, global cognitive function was assessed using the Mini-Mental State Examination (MMSE) [31,32]. An MMSE score of ≥24 was required for inclusion, consistent with MMSE eligibility criteria used in recent studies [33,34]. In the 19 older adults included in the primary cohort, the mean MMSE score was 27.47 (*SD* = 1.54; range = 24–30). Informed consent was obtained from all subjects before the experiment, and each participant received remuneration for their involvement afterward. The study was approved by the Ethics Committee of Tianjin Normal University, China.

### 2.2. Experimental Design

**Figure 1:**
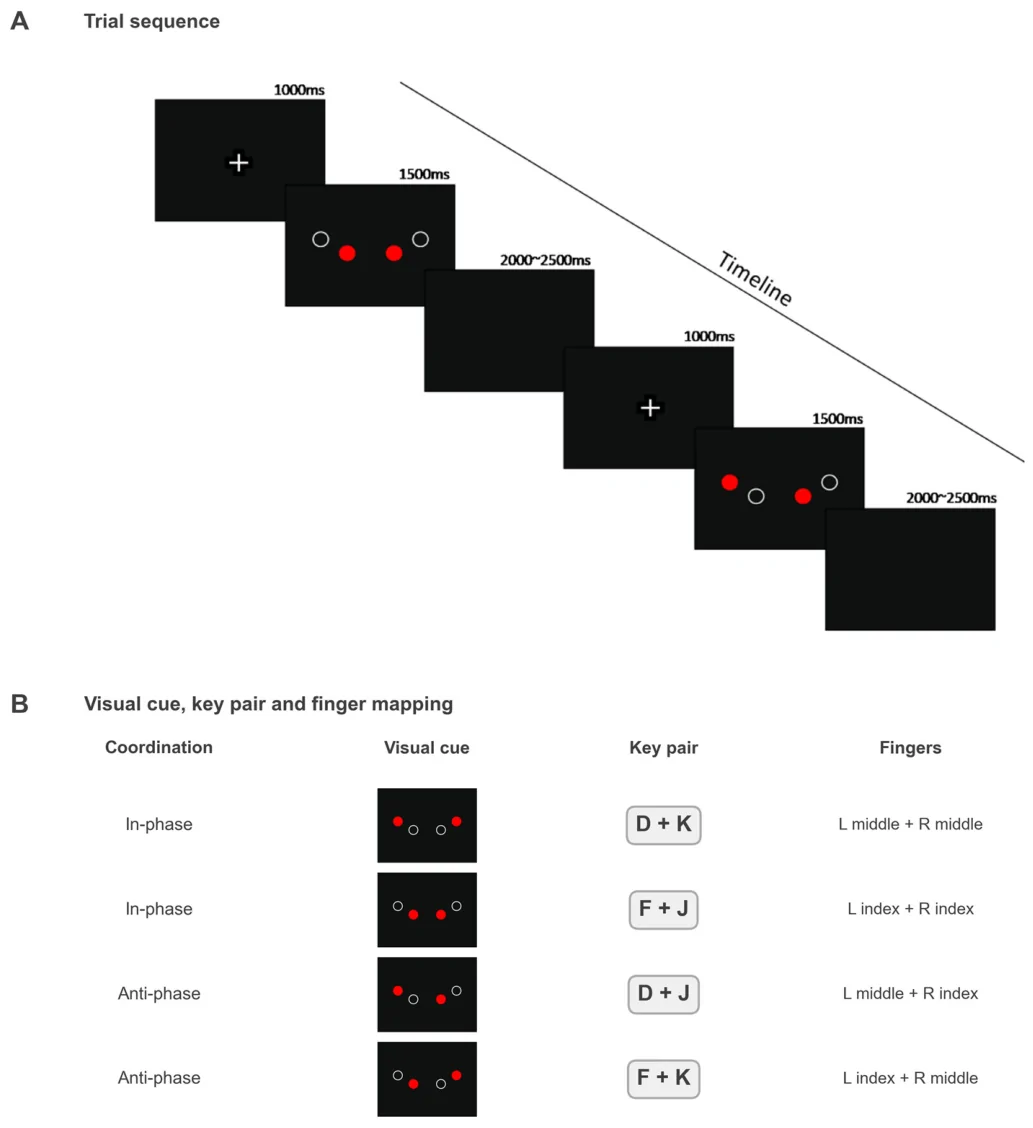
Schematic timeline of the modified choice-reaction bimanual coordination task. (**A**) Each trial began with a central fixation cross (1000 ms), followed by a visual target stimulus presented for up to 1500 ms or until a response was made. After the response (or after 1500 ms if no response), a blank screen was shown for 2000–2500 ms as an inter-trial interval. The four target configurations (D + K, F + J, D + J, and F + K) each appeared 40 times, yielding 160 trials in random order. (**B**) The four types of visual cues and the corresponding coordination mode, required key pair, and fingers. From left to right, the four circle positions corresponded to the D, F, J, and K keys, which were operated by the left middle, left index, right index, and right middle fingers, respectively. Red filled circles indicated the two keys to be pressed simultaneously; white open circles indicated the non-responding fingers. In-phase trials required symmetric finger pairs (D + K: left and right middle fingers; F + J: left and right index fingers), whereas anti-phase trials required asymmetric finger pairs (D + J: left middle and right index fingers; F + K: left index and right middle fingers). L/R: left/right hand. Before the formal experiment, participants practiced until reaching 80% accuracy.

The experiment used a mixed design that included two factors: age (young group and old group) and coordination mode (in-phase coordination and anti-phase coordination). The task was a modified choice-reaction task. The experiment was conducted using E-Prime 3.0, and the procedure is illustrated in Figure 1. The experiment began with a fixation point displayed in the center of the screen for 1000 ms to direct the participants’ attention. After this period, the target stimulus appeared on the screen for 1500 ms or until a response was made. Participants were instructed to press the corresponding buttons as quickly and accurately as possible when they saw the target stimulus. The target stimulus consisted of four horizontally arranged circles. From left to right, these positions corresponded to the D, F, J, and K keys, operated by the left middle, left index, right index, and right middle fingers, respectively. The two red filled circles indicated which two keys needed to be pressed simultaneously, while the white open circles represented the non-responding fingers. The positions of the red circles determined the required key pairs. As shown in Figure 1B, the in-phase target stimuli featured two symmetric cues, where participants pressed either the DK (using the left and right middle fingers) or FJ (using the left and right index fingers) buttons simultaneously. In contrast, the anti-phase target stimuli consisted of two asymmetric cues that required participants to press the DJ (with the left middle and right index fingers) or FK (with the left index and right middle fingers) buttons at the same time. Once the buttons were pressed, the target stimulus disappeared, and a blank screen was shown from 2000 to 2500 ms. Each of the four target configurations (DK, FJ, DJ, and FK) appeared 40 times, resulting in 160 trials presented in random order. Before the formal experiment, participants practiced until they reached an accuracy rate of 80%.

### 2.3. Behavioral Measures

The formal behavioral outcomes were accuracy, reaction time, and behavioral efficiency. A trial was scored as correct only when the required key pair was produced with an inter-press interval below 150 ms, allowing small timing differences between the hands while excluding markedly asynchronous responses [35,36]. Reaction time was defined as the latency of the second keypress, because the bimanual response was complete only when both keys had been registered. Only correct trials were included in the reaction time (RT) analyses. A lower RT cutoff of 100 ms was applied to screen for potentially anticipatory responses [37]; no trials fell below this threshold, and the remaining RTs were screened separately within each participant and coordination mode using a mean ± 3 *SD* criterion.

Subject-level mean RT and accuracy were computed for in-phase and anti-phase trials. Then participant-level mean RT was transformed using the natural logarithm (logRT). To summarize the joint cost of slowing and reduced accuracy, the inverse efficiency score (IES) was calculated for each participant and coordination mode as mean RT divided by the proportion of correct responses [38,39]. Smaller IES values indicate better performance efficiency: less time is required per correct response, which may reflect faster reaction times, higher accuracy, or both. Larger IES values indicate poorer performance efficiency: more time is required per correct response, which may reflect slower reaction times, lower accuracy, or both [38,39]. Because IES was positively skewed, log-transformed IES (logIES) was used as the primary behavioral-efficiency outcome in brain–behavior models.

### 2.4. EEG Data Acquisition and Preprocessing

The EEG data acquisition was carried out using the Neuroscan SynAmps 2 Curry 8 EEG signal acquisition system, which featured a 64-channel Ag/AgCl electrode cap according to the international 10–20 system. The default electrode reference point, located between the Cz and CPz electrodes, was used for data collection. Horizontal EOG electrodes were placed approximately 1 cm lateral to the outer canthus of each eye, and vertical EOG electrodes approximately 1 cm above and below the left eye. All electrode impedances were kept below 10 kΩ, and the data were sampled at a rate of 1000 Hz.

Preprocessing was carried out in MATLAB R2024a using EEGLAB [40]. Continuous data were band-pass filtered from 1 to 45 Hz and downsampled to 250 Hz. Artifact Subspace Reconstruction (ASR) was applied using the clean_rawdata plugin in EEGLAB, with the burst criterion set to 20 standard deviations [41]. Bad channels were identified using clean_rawdata with FlatlineCriterion = 5 s, ChannelCriterion = 0.85, and LineNoiseCriterion = 7, and were removed before independent component analysis (ICA). ICA was then performed using the Infomax algorithm. Artifactual components were removed if their ICLabel probabilities exceeded 0.90 for the ocular, cardiac, or myogenic classes. In the final analyzed sample, an average of 5.81 independent components per participant were removed (*SD* = 2.56; range = 1–11). After artifactual components had been removed, the previously excluded channels were restored by spherical spline interpolation, followed by global-average re-referencing. Stimulus-locked epochs extended from −1000 to 3000 ms relative to cue onset. Screening was performed automatically for each epoch, utilizing criteria based on variance, absolute amplitude, temporal gradient, and robust channel-aggregated peak-to-peak values [42,43,44]. Epochs were rejected if peak-to-peak amplitude exceeded 250 µV, if at least 30% of channels were nearly flat, or if at least two robust artifact metrics exceeded |robust z| > 4. Participants were retained for EEG analyses only when at least 30 artifact-free epochs remained in each coordination mode.

### 2.5. ERP Analysis

**Figure 2:**
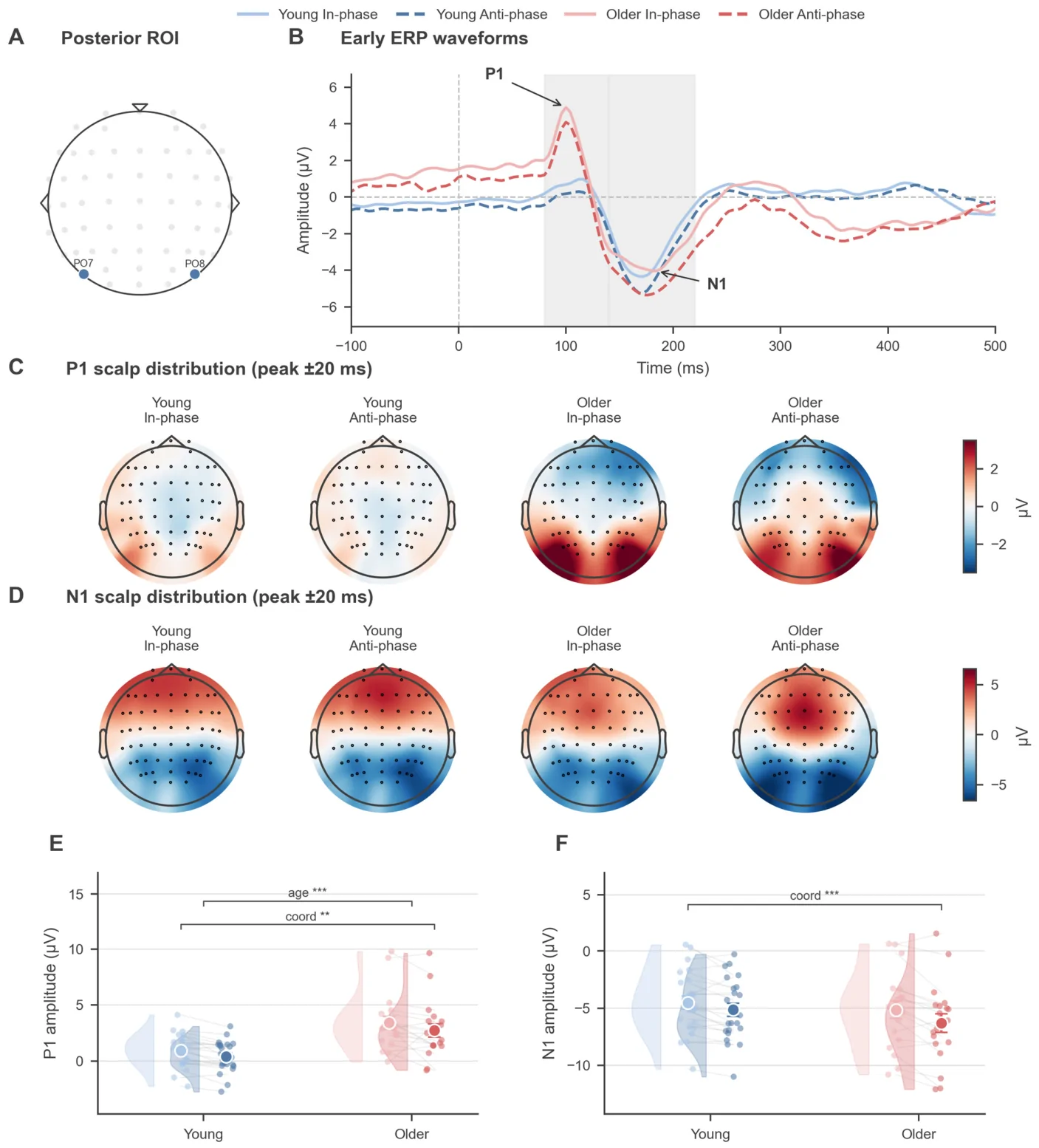
(**A**) Posterior ROI (PO7/PO8). (**B**) Grand-average waveforms with P1 (80–140 ms) and N1 (140–220 ms) search windows shaded. (**C**,**D**) P1 and N1 topographies, averaged within ±20 ms of each participant’s condition-specific posterior peak. (**E**,**F**) Individual amplitudes and means ± *SEM*; lines connect paired conditions. Blue/red indicate younger/older adults; light/dark shades and solid/dashed waveforms indicate in-phase/anti-phase conditions. *N* = 42. “age” and “coord” denote main effects. * *q* < 0.05, ** *q* < 0.01, *** *q* < 0.001. Stars denote BH-FDR-adjusted significance.

All ERP analyses used a common prestimulus baseline of −800 to −300 ms. Early components (P1 and N1 components) were averaged across PO7 and PO8 from a posterior ROI (Figure 2A). Peak detection was automated: P1 was defined as the maximum within 80–140 ms, and N1 as the minimum within 140–220 ms. Savitzky–Golay smoothing (20 ms window, five samples at 250 Hz, third-order polynomial) was applied only for peak detection. Amplitudes were then measured from the unsmoothed waveform as the mean within ±20 ms of each participant’s ROI-averaged peak, and latency was defined as the corresponding peak latency. No minimum peak-width criterion was imposed; boundary peaks and low-amplitude or low-prominence peaks were flagged for quality review rather than automatically excluded.

**Figure 3:**
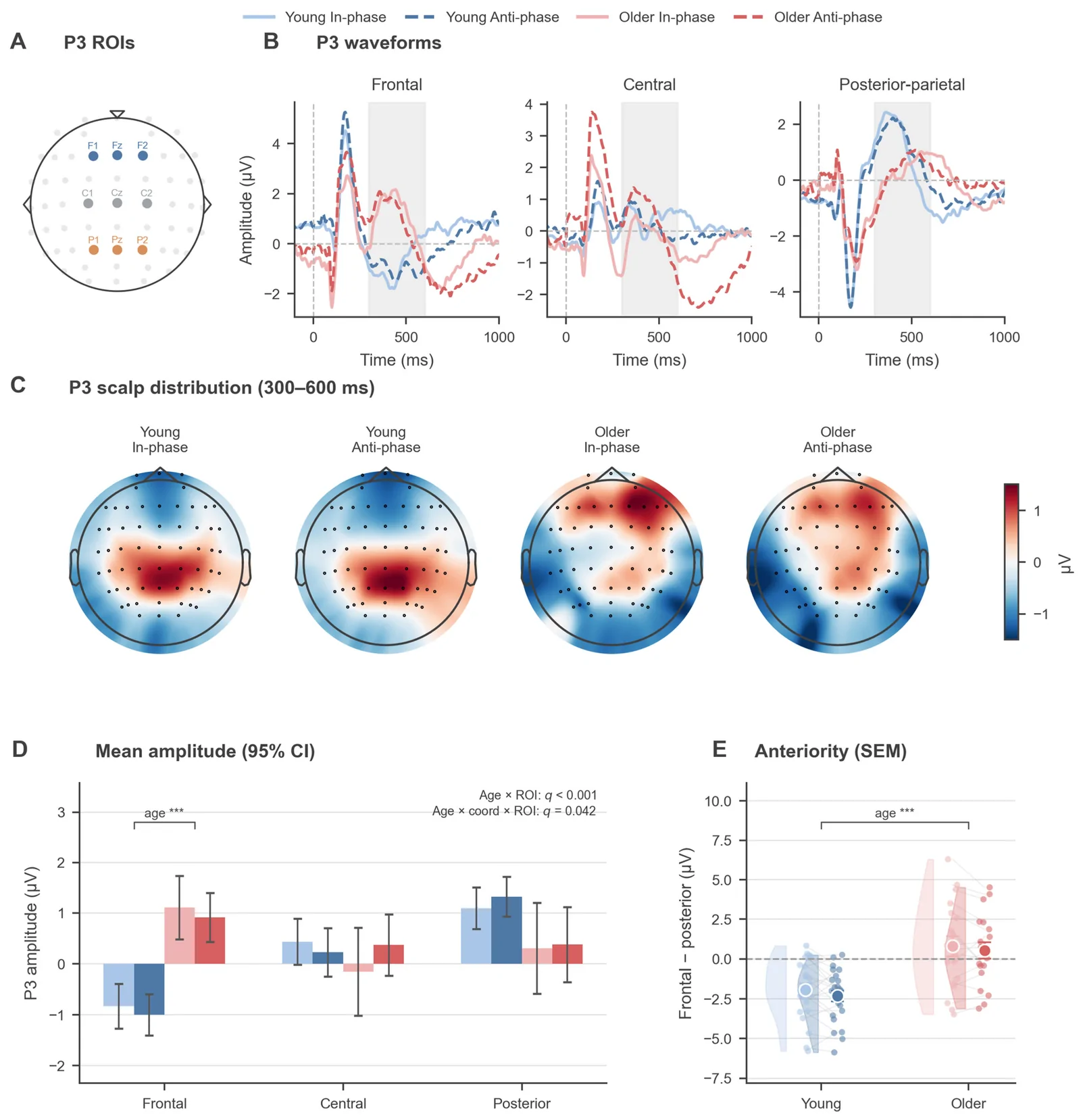
Age-related sensor-level organization of the P3 response. (**A**) Frontal, central, and posterior-parietal ROIs. (**B**) Grand-average waveforms; shading marks 300–600 ms time window. (**C**) Mean-amplitude topographies for 300–600 ms time window. (**D**) ROI amplitudes, mean ± 95% CI. (**E**) Anteriority index (frontal minus posterior-parietal amplitude), individual values and mean ± *SEM*. Colors and line styles follow Figure 2. *N* = 42. The frontal age contrast in (**D**) and the age effect in (**E**) are Holm-adjusted; the omnibus tests in (**D**) use BH-FDR correction. Stars denote corrected *p* or *q* < 0.05 (*), 0.01 (**), and 0.001 (***). The topographies and ROI contrasts describe sensor-level distributions and do not identify cortical generators.

Meanwhile, late positive activity (P3 component) was quantified from three a priori ROIs: frontal (F1, Fz, F2), central (C1, Cz, C2), and posterior-parietal (P1, Pz, P2) (Figure 3A). These ROIs sampled the anterior–posterior distribution of P3 activity, consistent with frontal, central, and parietal comparisons in recent aging studies [45,46]. Each ROI combined a midline electrode with its two adjacent lateral electrodes. The primary P3 amplitude measure was the mean amplitude in the 300–600 ms window. This choice was guided by methodological work showing that mean-amplitude measures are less susceptible than local peak measures to background noise, particularly when waveform morphology and latency jitter vary across participants or groups [47]. To further assess the spatial shift in late positive activity, we computed a P3 anteriority index from P3 amplitudes, which was defined as the frontal P3 amplitude minus posterior-parietal amplitude. A higher value of the index indicates a more anterior distribution of P3 activity, whereas a lower value indicates a more posterior-parietal distribution.

P3 latency was quantified as 50% fractional-area latency within a 300–650 ms search window. Area-based latency measures are less dependent on the definition of a single local peak and are well-suited to broad ERP components [48]. Peak-centered ±25 ms amplitude and peak latency were also computed as comparison analyses.

### 2.6. Time–Frequency Analysis

Time–frequency decomposition was performed on stimulus-locked epochs using complex Morlet wavelets from 4 to 30 Hz in 1 Hz steps. The number of cycles increased with frequency and was defined as max(f/2, 3), allowing wavelet length to scale with frequency. Analyses focused on beta-band power (13–30 Hz), which was used to characterize movement-related desynchronization and rebound-like activity [21,22]. For each participant, coordination mode, and ROI, beta power was extracted from the same frontal (F1/Fz/F2), central (C1/Cz/C2), and posterior-parietal (P1/Pz/P2) ROIs used for the P3 analyses.

Prestimulus beta power was quantified as absolute log_10_-transformed beta power from −800 to −300 ms relative to cue onset. Relative beta power was expressed in decibels as 10 × log_10_ (current beta power/mean prestimulus beta power). Values below 0 dB indicated beta suppression relative to the prestimulus interval. Absolute beta power was retained in parallel analyses to evaluate whether fixed-window effects remained after adjustment for prestimulus beta variation.

To test the robustness of time–frequency differences across groups and conditions, three time–frequency measures were analyzed. First, mean relative beta power was extracted from a window of 100–500 ms, which was intended to capture cue-related response selection and movement-preparatory beta dynamics [21,22]. Second, mean absolute log_10_ beta power was extracted from the same 100–500 ms window. Third, cumulative ERD burden was calculated from individualized operational ERD episodes.

Individualized operational ERD episodes were identified for each participant, condition, and ROI. At each poststimulus time point, relative beta power was tested against 0 dB using one-sample permutation tests with 10,000 permutations. Candidate ERD episodes were defined as runs of at least five consecutive time points meeting the corresponding pointwise *p* < 0.05 criterion. When multiple candidate ERD periods were present, the primary episode was selected by prioritizing overlap with the 100–500 ms early beta window. Later ERD segments were merged forward only when the intervening gap was no longer than 80 ms. Onset, offset, and duration were treated as operational descriptors of the detected ERD episode rather than precise neurophysiological timing estimates. Cumulative ERD burden was calculated by integrating the magnitude of negative baseline-relative beta-power value over each identified ERD episode using the trapezoidal rule and was expressed in dB·ms. Related approaches have quantified ERD using the area under the ERD curve [25] or cumulative sums of ERD/ERS waveforms [49]. Larger values indicate stronger beta suppression, longer duration, or both. When no ERD episode was detected, the value was treated as missing rather than zero.

### 2.7. Statistical Analysis

Behavioral accuracy, RT, logRT, raw IES, and logIES were analyzed using 2 × 2 mixed-design models with age group as the between-participant factor and coordination mode as the within-participant factor. Notably, the primary behavioral analyses used the 42-participant EEG-aligned sample. The same models were repeated in all 45 enrolled participants as supplementary robustness analyses.

Early ERP measures were analyzed with separate 2 × 2 mixed ANOVAs. P3 amplitude and latency were analyzed with 2 × 2 × 3 mixed ANOVAs including ROI as a within-participant factor. The P3 anteriority index was analyzed with a 2 × 2 mixed ANOVA. Prestimulus beta power was analyzed with an age group × ROI mixed ANOVA.

Multiplicity across ERP endpoints was controlled using the Benjamini–Hochberg FDR procedure, applied separately to each design effect across the four early ERP measures and the four P3 measures. For ROI-related P3 effects, Greenhouse–Geisser-corrected *p* values entered the FDR correction. The three P3 anteriority effects were adjusted separately using Holm’s procedure. Follow-up contrasts were performed only for omnibus effects surviving FDR correction (*q* < 0.05) and were Holm-adjusted. Accordingly, omnibus results are reported as q values, whereas anteriority and follow-up results are reported as Holm-adjusted *p* values.

The three retained time–frequency metrics (relative beta power, absolute beta power, and cumulative ERD burden) were analyzed separately using ROI-factor linear mixed-effects models. For relative beta power and cumulative ERD burden, the model was:

Metric ~ Age Group × Coordination Mode × ROI + (1|participant) + (1|participant:ROI).

For absolute beta power, the corresponding centered prestimulus beta-power value and its interaction with ROI were included as nuisance covariate terms:

Absolute beta power ~ Age Group × Coordination Mode × ROI + prestimulus beta power × ROI + (1|participant) + (1|participant: ROI).

The participant random intercept accounted for repeated observations within participants, and the participant-by-ROI random intercept accounted for participant-specific ROI baselines and repeated observations within each participant–ROI combination. Fixed effects in the linear mixed-effects models were evaluated using Type III F tests with Satterthwaite-approximated degrees of freedom. Continuous covariates were centered before entry into the time–frequency models. Missing ERD-burden values were not imputed or replaced with zero; models involving ERD burden used all available non-missing observations.

For each metric, the seven fixed-effect terms representing the experimental design—three main effects, three two-way interactions, and one three-way interaction—were included in a single 21-test family across the three metrics. Multiple comparisons within this family were controlled using the Benjamini–Hochberg false discovery rate procedure. The prestimulus beta-power main effect and Prestimulus Beta Power × ROI interaction were treated as nuisance-control terms and were excluded from both the FDR family and the ROI-gating decision.

ROI-separated follow-up models were fitted only when the ROI main effect or at least one experimental-design interaction involving ROI survived FDR correction at *q* < 0.05 in the full ROI-factor model. Within each ROI, the following model was fitted:

Metrics ~ Age Group × Coordination Mode + (1|participant).

The corresponding absolute-power models additionally included the matched prestimulus beta-power value as a covariate. ROI-separated follow-up tests were adjusted using the Benjamini–Hochberg procedure within each prespecified endpoint-specific family. A significant effect within an individual ROI was not interpreted as evidence of ROI specificity. Evidence for coordination-related ROI specificity required an FDR-corrected Coordination Mode × ROI or Age Group × Coordination Mode × ROI effect in the full ROI-factor model.

Within each ROI, coordination contrasts were evaluated only when the corresponding Age Group × Coordination Mode interaction survived correction. For each endpoint, the six age-by-ROI contrasts were adjusted using the Benjamini–Hochberg procedure (Table S2). Statistical significance was defined as *α* = 0.05, and all coefficient and contrast tests were two-sided.

### 2.8. Brain–Behavior Analyses

Exploratory brain–behavior analyses examined logIES and logRT separately. Natural-log transformations reduced right skew and expressed associations on a proportional scale. Five neural features were tested for each outcome: P3 anteriority, posterior-parietal P3 50% fractional-area latency, cumulative ERD burden, relative beta power in the 100–500 ms window, and absolute beta power in the same window. For the beta-band features, ROI-averaged values were used to provide condition-level neural summaries; relative beta power and absolute beta power were averaged across the frontal, central, and posterior-parietal ROIs. Cumulative ERD burden was derived from the ROI-integrated time course rather than averaged from separate ROI burdens; observations without a detected episode were treated as missing. Missing ERD-burden values were not imputed or replaced with zero. Each neural feature was z-standardized within its corresponding analytic dataset before model fitting.

For P3 anteriority, posterior-parietal P3 50% fractional-area latency, ERD burden, and relative beta power, the following single-feature model was fitted:

logIES/logRT ~ Age Group × Coordination Mode + z(neural feature) + (1|participant),

For absolute beta power, the corresponding condition-matched prestimulus beta-power value was also z-standardized and included as a nuisance covariate:

logIES/logRT ~ Age Group × Coordination Mode + z(absolute beta power) + z(prestimulus beta power) + (1|participant).

For each behavioral outcome, the neural-feature coefficients from the five separate models formed a five-test family controlled using the Benjamini–Hochberg false discovery rate procedure. Notably, missing ERD-burden values were neither imputed nor replaced with zero, and each model reported the number of participants contributing data.

## 3. Results

### 3.1. Behavioral Performance

In the primary cohort (*N* = 42), the older adults were less accurate overall, *F*(1, 40) = 9.54, *p* = 0.004, η^2^_G_ = 0.156, and accuracy was lower in the anti-phase than in-phase condition, *F*(1, 40) = 35.96, *p* < 0.001, η^2^_G_ = 0.168. These effects were qualified by an Age Group × Coordination interaction, *F*(1, 40) = 18.54, *p* < 0.001, η^2^_G_ = 0.094, reflecting a larger age-group difference in the anti-phase condition.

Raw RT showed significant effects of Age Group, *F*(1, 40) = 53.18, *p* < 0.001, η^2^_G_ = 0.536, Coordination Mode, *F*(1, 40) = 198.03, *p* < 0.001, η^2^_G_ = 0.393, and their interaction, *F*(1, 40) = 25.43, *p* < 0.001, η^2^_G_ = 0.077. The same pattern was observed for logRT, with significant effects of Age Group, *F*(1, 40) = 54.52, *p* < 0.001, η^2^_G_ = 0.554, Coordination Mode, *F*(1, 40) = 275.15, *p* < 0.001, η^2^_G_ = 0.383, and their interaction, *F*(1, 40) = 13.60, *p* < 0.001, η^2^_G_ = 0.030. Holm-adjusted contrasts confirmed that older adults were slower in both conditions, anti-phase responses were slower in both age groups, and the age-group difference was larger in the anti-phase condition (all adjusted *p* < 0.001). Descriptive values are presented in Table 1.

IES and logIES showed the same pattern [IES: Age Group, *F*(1, 40) = 29.91, *p* < 0.001, η^2^_G_ = 0.359; Coordination, *F*(1, 40) = 59.59, *p* < 0.001, η^2^_G_ = 0.273; interaction, *F*(1, 40) = 16.78, *p* < 0.001, η^2^_G_ = 0.096; logIES: Age Group, *F*(1, 40) = 45.29, *p* < 0.001, η^2^_G_ = 0.494; Coordination, *F*(1, 40) = 166.83, *p* < 0.001, η^2^_G_ = 0.363; interaction, *F*(1, 40) = 20.46, *p* < 0.001, η^2^_G_ = 0.065]. The supplementary analyses of all 45 recruited participants yielded the same qualitative pattern and the same inferential conclusions for accuracy, logRT, IES, and logIES (Table S1).

### 3.2. ERP Results

#### 3.2.1. Early Activity

ERP analyses included 42 participants (23 younger and 19 older adults). P1 amplitude was larger in older than younger adults, *F*(1, 40) = 16.35, *p* < 0.001, *q* < 0.001, η^2^_G_ = 0.275, and larger in the in-phase than anti-phase condition, *F*(1, 40) = 13.52, *p* < 0.001, *q* = 0.001, η^2^_G_ = 0.024. The Age Group × Coordination Mode interaction was not significant, *F*(1, 40) = 0.24, *p* = 0.629, *q* = 0.936. Holm-corrected contrasts confirmed the age-group difference, *t*(40) = 4.04, Holm-adjusted *p* < 0.001, and the coordination-mode difference, *t*(40) = 3.68, Holm-adjusted *p* < 0.001.

N1 amplitude was more negative in the anti-phase than in-phase condition, *F*(1, 40) = 26.44, *p* < 0.001, *q* < 0.001, η^2^_G_ = 0.021. Neither the age-group main effect, *F*(1, 40) = 0.92, *q* = 0.458, nor the Age Group × Coordination Mode interaction, *F*(1, 40) = 2.43, *q* = 0.509, was significant. The Holm-corrected coordination contrast was significant, *t*(40) = 5.14, Holm-adjusted *p* < 0.001. No P1- or N1-latency effect survived FDR correction (all *q* ≥ 0.458). The P1 and N1 waveforms, as well as the statistical results for P1 and N1 amplitudes, are presented in Figure 2B, Figure 2E, Figure 2F, respectively.

#### 3.2.2. P3 Component

Mean P3 amplitude in the 300–600 ms window showed an Age Group × ROI interaction, *F*(1.52, 60.90) = 15.92, *p* < 0.001, *q* < 0.001, η^2^_G_ = 0.194, and an Age Group × Coordination Mode × ROI interaction, *F*(1.46, 58.45) = 5.75, *p* = 0.011, *q* = 0.042, η^2^_G_ = 0.006. The age-group, coordination-mode, and Age Group × Coordination Mode effects were not significant after FDR correction (all *q* ≥ 0.323). The ROI main effect and Coordination Mode × ROI interaction each reached *q* = 0.052. The mean amplitude of P3 across the three ROIs and the scalp topographies of mean P3 amplitude are presented in Figure 3D and Figure 3C, respectively.

Holm-corrected decomposition of the three-way interaction showed that, in younger adults, frontal amplitude was lower than central amplitude during both anti-phase coordination, *t*(40) = −4.52, Holm-adjusted *p* = 0.001, and in-phase coordination, *t*(40) = −4.14, Holm-adjusted *p* = 0.004. Frontal amplitude was also lower than posterior-parietal amplitude in the anti-phase condition, *t*(40) = −5.68, Holm-adjusted *p* < 0.001, and in-phase condition, *t*(40) = −3.97, Holm-adjusted *p* = 0.005. Central amplitude was lower than posterior-parietal amplitude only in the younger anti-phase condition, *t*(40) = −3.80, Holm-adjusted *p* = 0.009. In older adults, frontal amplitude exceeded central amplitude in the in-phase condition, *t*(40) = 3.75, Holm-adjusted *p* = 0.010; no ROI contrast survived correction in the older anti-phase condition. Frontal amplitude was larger in older than younger adults in both coordination modes, |*t*(40)| ≥ 5.41, both Holm-adjusted *p* < 0.001. No corrected age-group difference was present in the central or posterior-parietal ROI, and no within-age, within-ROI coordination contrast survived correction (all Holm-adjusted *p* ≥ 0.068) (for details, see Figure 3D).

The P3 anteriority index showed a main effect of age group, *F*(1, 40) = 18.67, *p* < 0.001, Holm-adjusted *p* < 0.001, η^2^_G_ = 0.304, with higher values in older than younger adults. Neither the coordination-mode main effect, *F*(1, 40) = 3.99, *p* = 0.053, Holm-adjusted *p* = 0.105, nor the Age Group × Coordination Mode interaction, *F*(1, 40) = 0.17, *p* = 0.686, Holm-adjusted *p* = 0.686, was significant (see Figure 3E). Overall, these findings suggest a more anterior scalp distribution among older adults.

For P3 50% fractional-area latency, in-phase trials had longer latencies than anti-phase trials, *F*(1, 40) = 11.72, *p* = 0.001, *q* = 0.003, η^2^_G_ = 0.014. The Age Group × ROI interaction, *F*(1.43, 57.28) = 12.67, *p* < 0.001, *q* < 0.001, η^2^_G_ = 0.158, and Coordination Mode × ROI interaction, *F*(1.93, 77.17) = 8.58, *p* < 0.001, *q* = 0.002, η^2^_G_ = 0.019, were significant. The age-group and ROI main effects and the three-way interaction were not significant (all *q* ≥ 0.373). Relative to younger adults, older adults had a 48.15 ms shorter frontal latency, *t*(40) = 3.01, Holm-adjusted *p* = 0.032, and a 53.51 ms longer posterior-parietal latency, *t*(40) = 3.78, Holm-adjusted *p* = 0.005; the central age-group contrast was not significant. Within younger adults, frontal latency exceeded posterior-parietal latency by 52.61 ms, *t*(40) = 2.97, Holm-adjusted *p* = 0.032. Within older adults, posterior-parietal latency exceeded central latency by 45.79 ms, *t*(40) = 3.07, Holm-adjusted *p* = 0.030. The coordination-mode contrast was confined to the central ROI, where in-phase latency exceeded anti-phase latency by 28.01 ms, *t*(40) = 3.88, Holm-adjusted *p* = 0.003.

### 3.3. Time–Frequency Analysis Results

#### 3.3.1. Prestimulus Beta Baseline

Prestimulus absolute beta power was higher in older than younger adults, *F*(1, 40) = 18.01, *q* < 0.001, η^2^_G_ = 0.294, and differed across ROIs, *F*(1.81, 72.44) = 48.15, *q* < 0.001, η^2^_G_ = 0.083. The Age Group × ROI interaction was significant, *F*(1.81, 72.44) = 8.56, *q* = 0.002, η^2^_G_ = 0.016. Holm-corrected comparisons showed higher prestimulus beta power in older adults in the frontal, central, and posterior-parietal ROIs (all adjusted *p* ≤ 0.012).

#### 3.3.2. Fixed-Window Beta Suppression

In the ROI-factor model for relative beta power, older adults showed more negative 100–500 ms beta values than younger adults, *F*(1, 42.00) = 30.72, *q* < 0.001. Relative beta power also differed across ROIs, *F*(2, 84.00) = 30.95, *q* < 0.001. The Age Group × ROI interaction did not survive correction, *q* = 0.074, and no coordination-related omnibus effect or interaction was significant.

**Figure 4:**
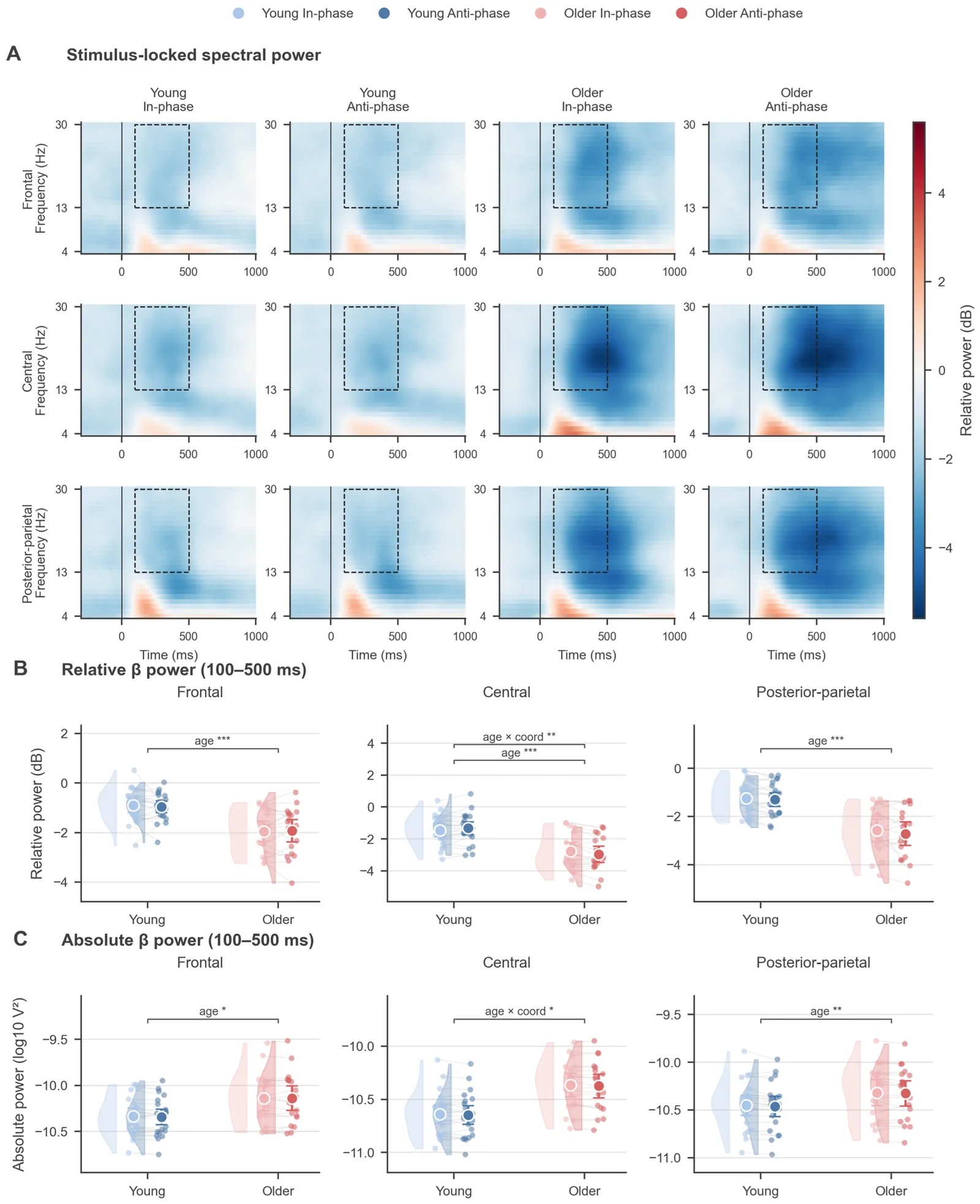
Time–frequency responses and fixed-window beta power. (**A**) ROI time–frequency maps on a common color scale relative to the −800 to −300 ms baseline; rectangles mark 13–30 Hz and 100–500 ms. (**B**,**C**) Relative and absolute beta power: individual values, paired conditions, and means with 95% CIs. Absolute-power values are unadjusted; tests control for prestimulus power. “age” and “age × coord” indicate BH-FDR-adjusted effects. Corrected significance: * *q* < 0.05, ** *q* < 0.01, *** *q* < 0.001.

The corrected ROI effect opened the conditional ROI analyses. Age-group effects were observed in the frontal ROI, *F*(1, 42.00) = 21.75, *q* < 0.001, central ROI, *F*(1, 42.00) = 24.43, *q* < 0.001, and posterior-parietal ROI, *F*(1, 42.00) = 31.80, *q* < 0.001. The central ROI additionally showed an Age Group × Coordination Mode interaction, *F*(1, 42.00) = 9.74, *q* = 0.007, reflecting a larger age-group difference during anti-phase coordination. Because the Age Group × Coordination Mode × ROI interaction was not significant in the full model, *q* = 0.128, this follow-up does not establish a central-specific coordination effect (for details, see Figure 4B).

The baseline-adjusted absolute-power model showed effects of age group, *F*(1, 49.05) = 7.99, *q* = 0.016, and ROI, *F*(2, 81.97) = 487.37, *q* < 0.001. After adjustment for matched prestimulus beta power, older adults showed lower task-period absolute beta power. No experimental-design interaction involving ROI survived correction.

Conditional ROI analyses showed age-group effects in the frontal ROI, *F*(1, 41.48) = 6.83, *q* = 0.037, and posterior-parietal ROI, *F*(1, 41.77) = 15.69, *q* = 0.003. The central ROI showed an Age Group × Coordination Mode interaction, *F*(1, 42.77) = 6.95, *q* = 0.037. However, the full Age Group × Coordination Mode × ROI interaction was not significant, *q* = 0.148. The central follow-up therefore cannot be interpreted as formal evidence of coordination-related ROI specificity. The absolute beta power in young and older adults across three ROIs was shown in Figure 4C.

#### 3.3.3. Individualized ERD Suppression and Cumulative Burden

Cumulative ERD burden was available for 41 participants, with undetected episodes retained as missing. In the ROI-factor model, ERD burden showed effects of age group, *F*(1, 41.80) = 54.60, *q* < 0.001, coordination mode, *F*(1, 112.66) = 68.09, *q* < 0.001, ROI, *F*(2, 79.16) = 26.97, *q* < 0.001, Age Group × Coordination Mode, *F*(1, 112.66) = 64.35, *q* < 0.001, and Age Group × ROI, *F*(2, 79.16) = 10.30, *q* < 0.001. The Coordination Mode × ROI interaction, *q* = 0.128, and three-way interaction, *q* = 0.148, were not significant.

**Figure 5:**
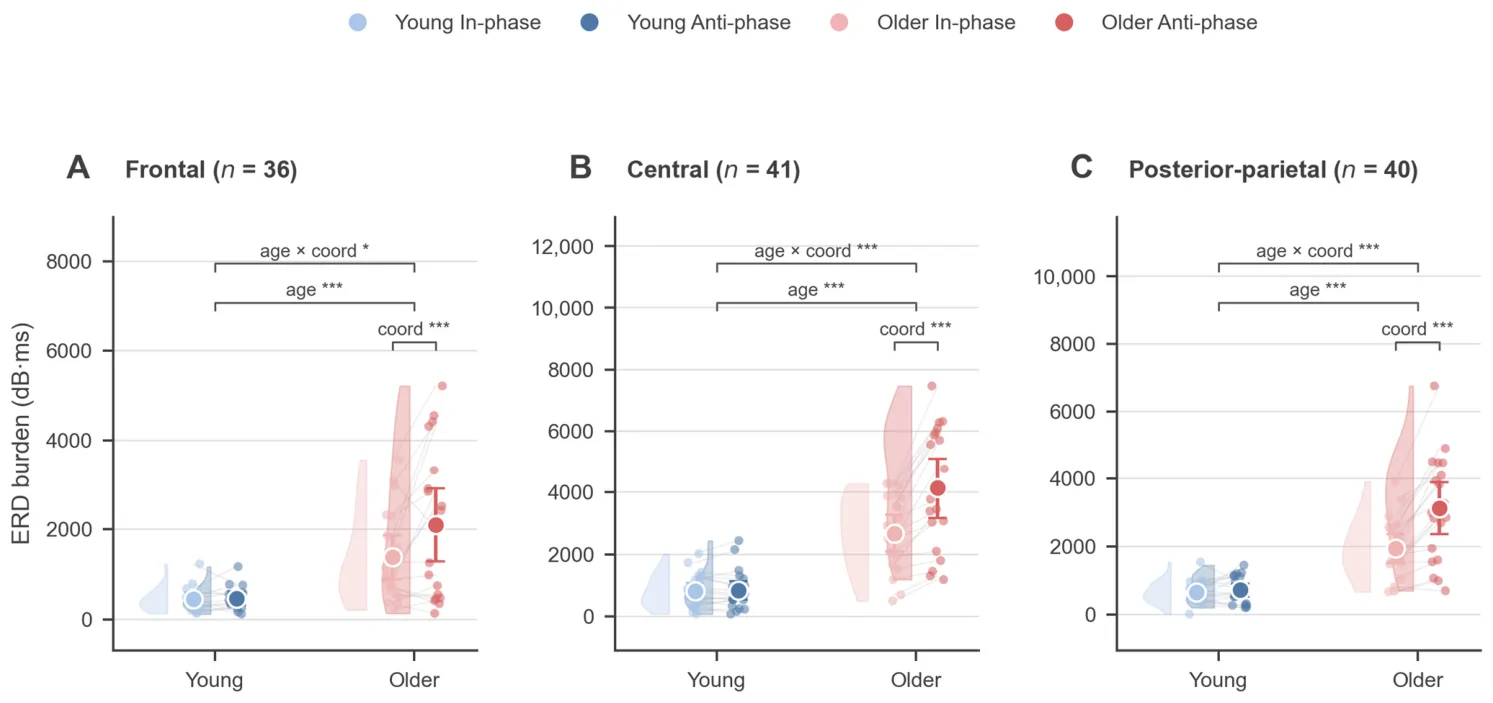
Cumulative beta ERD burden across regions. (**A**–**C**) Frontal, central, and posterior-parietal burden, respectively, defined as integrated beta suppression over the identified ERD episode. Small points show individual values, lines connect paired conditions, and large symbols show means with 95% CIs. Valid observations come from 36, 41, and 40 participants, respectively. “age” and “age × coord” denote age-group and interaction effects; “coord” denotes the within-older-group coordination contrast. Significance levels are based on BH-FDR-adjusted *q* values: * *q* < 0.05, ** *q* < 0.01, *** *q* < 0.001.

Conditional ROI analyses included 36 participants in the frontal ROI, 41 in the central ROI, and 40 in the posterior-parietal ROI. Age-group, coordination-mode, and Age Group × Coordination Mode effects survived correction in all three ROIs. Across ROIs, the greatest ERD burden occurred in older adults during anti-phase coordination. Because neither the Coordination Mode × ROI nor the three-way interaction was significant in the full model, the coordination effect was interpreted as broadly expressed across the three ROIs rather than ROI-specific. For individualized cumulative event-related desynchronization burden across ROIs and the full statistical results, please refer to Figure 5 and Table S2.

#### 3.3.4. Brain–Behavior Associations

**Figure 6:**
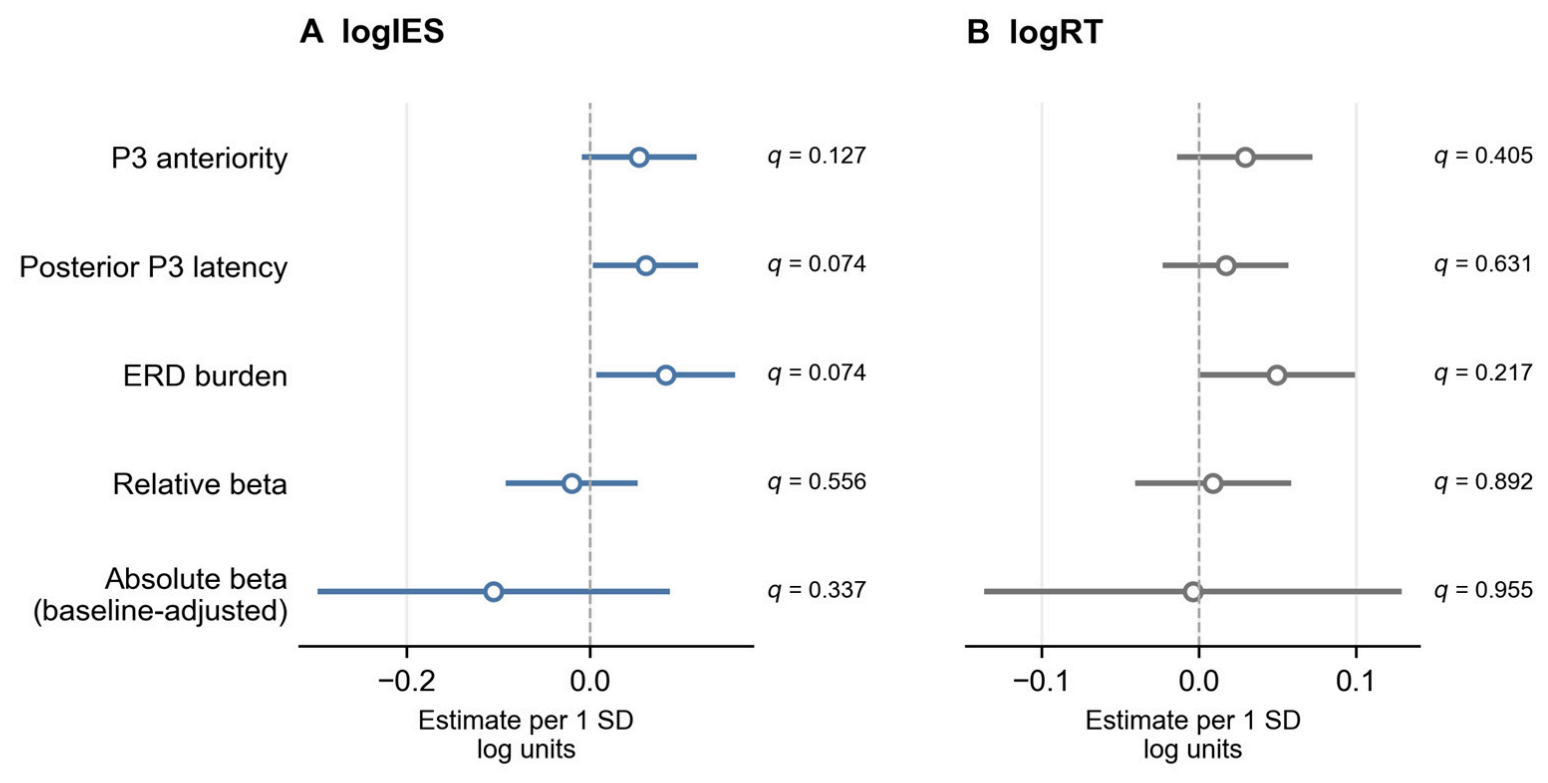
Exploratory brain–behavior analyses. Forest plots show design-adjusted associations of five neural measures with (**A**) log-transformed inverse efficiency score (logIES) and (**B**) log-transformed reaction time (logRT). Points and horizontal lines represent regression estimates per 1-*SD* increase in each neural measure and 95% confidence intervals. Models include age group, coordination mode, their interaction, and a participant random intercept; absolute beta additionally adjusts for condition-matched prestimulus beta power. BH-FDR-adjusted *q* values are shown beside the estimates, with separate five-test families for the two outcomes. Burden models include 41 participants; the other models include 42.

None of the five neural features were associated with logIES after correction across the five-feature family (Figure 6 and Table S3). Posterior-parietal P3 latency showed a positive nominal association with logIES, *β* = 0.060 per 1 *SD*, 95% CI [0.006, 0.115], *p* = 0.030, *q* = 0.074, as did ERD burden, *β* = 0.082, 95% CI [0.010, 0.155], *p* = 0.027, *q* = 0.074. No neural feature was associated with logRT after correction. ERD burden showed a nominal positive association, *β* = 0.050 per 1 *SD*, 95% CI [0.002, 0.098], *p* = 0.043, *q* = 0.217. 

## 4. Discussion

The present study investigated age-related differences in bimanual coordination at behavioral, ERP, and oscillatory levels. The results indicated that age-related differences were present in ERP responses and beta-band dynamics, with behavioral impairments most pronounced in the anti-phase condition. Among the beta measures, only cumulative ERD burden exhibited a corrected Age Group × Coordination Mode interaction, driven by the greatest burden in older adults during anti-phase coordination. The exploratory analyses provided no FDR-corrected evidence of associations with logIES or logRT, leaving their functional relationship unresolved. Therefore, the observed age effects may reflect age-related differences in task-related neural processing across multiple levels, although the present measures cannot isolate distinct processing stages or rule out contributions from response-related activity.

The behavioral findings demonstrated age-related declines in bimanual coordination performance. Older adults exhibited longer reaction times than younger adults across both coordination modes, with the largest age-related difference observed during anti-phase coordination. In addition, while accuracy was comparable between groups during the relatively simple in-phase task, older adults showed significantly reduced accuracy during anti-phase coordination. These results are consistent with previous evidence indicating that age-related motor deficits become increasingly pronounced as task complexity rises [5,6,7,23]. Importantly, the greater performance cost observed in the anti-phase condition suggests that aging disproportionately affects the ability to perform cognitively demanding forms of coordination. Compared with in-phase coordination, anti-phase coordination is inherently less stable and requires greater executive control to maintain the intended movement pattern and suppress the tendency toward more stable coordination modes. Therefore, the marked slowing and reduced accuracy observed in older adults likely reflect the combined influence of increased interlimb coordination demands, response-selection demands, and age-related changes in cognitive–motor control, rather than coordination difficulty alone [8,50]. Together, these findings suggest that age-related deterioration in higher-order cognitive control mechanisms may be a key factor underlying impairments in complex bimanual coordination.

The P1 component reflects early visual sensory encoding and attentional modulation of stimulus features, with major neural generators located in extrastriate visual areas and maximal activity typically observed over occipital regions [18]. In the present study, older adults showed larger P1 amplitudes than younger adults, consistent with previous findings of enhanced early visual ERP responses in aging [51,52]. This age-related P1 enhancement suggests that older adults may allocate more neural resources during early perceptual processing. While this could help support subsequent perceptual-motor processing, it may also reflect reduced neural efficiency or decreased inhibitory control with aging. P1 amplitude was also larger in the in-phase than in the anti-phase condition. This suggests that the two coordination modes were already differentiated at an early perceptual stage. The stronger P1 response to in-phase cues may reflect the greater perceptual compatibility or familiarity of in-phase coordination, which is generally more stable and easier to process than anti-phase coordination.

The N1 component is associated with early attentional selection and perceptual discrimination, typically occurring around 100–200 ms after stimulus onset [53]. In contrast to P1, N1 amplitude was more negative in the anti-phase than in the in-phase condition, while no significant age effect or Age Group × Coordination interaction was found. This pattern suggests that anti-phase coordination required greater early attentional discrimination or perceptual–motor mapping demands in both age groups. Importantly, neither P1 nor N1 latency showed significant age-related differences, indicating that aging did not significantly delay early visual cue processing in the present task. Instead, the main age-related change at this early stage was reflected in the magnitude of neural activation, particularly the enhanced P1 amplitude in older adults.

Together, early ERP analyses suggest that early visual processing of bimanual coordination cues is influenced by both aging and coordination mode, but in different ways. Aging was mainly associated with increased early sensory-perceptual activation, whereas coordination mode modulated both P1 and N1 amplitudes, indicating that in-phase and anti-phase cues were distinguished during early perceptual and attentional processing stages.

For the P3 component, no overall age-group effect on amplitude or latency survived FDR correction; nevertheless, older adults showed larger frontal amplitudes and a higher anteriority index than younger adults, consistent with a more anteriorly weighted scalp distribution of late positive activity. P3 amplitude is generally considered an index of attentional resource allocation and context updating [19,54], whereas P3 latency reflects the timing of stimulus evaluation and categorization processes [55,56]. Importantly, frontal P3a activity has been linked to attentional orienting and cognitive control, whereas posterior P3b activity is more strongly associated with attentional allocation and context updating [57]. In the present study, younger adults displayed the typical posterior-dominant P3 distribution, whereas older adults showed enhanced frontal positivity and a significantly higher anteriority index. Moreover, the latency pattern was also redistributed, with earlier frontal and delayed posterior-parietal P3 activity in older adults. These findings suggest that aging alters the topographical organization of P3-related processing rather than uniformly reducing its magnitude. Regional latency differences may partly reflect overlapping component contributions at the fixed ROIs [58].

This pattern is consistent with previous reports showing that cognitively healthy older adults can exhibit preserved P3 responses accompanied by a more anterior scalp distribution [59,60]. Alperin and colleagues further proposed that increased anterior P3a-like activity may temporally overlap with posterior P3b activity in aging, resulting in an apparent anterior shift of the P3 complex [58,61]. Frontal P3 predominance has also been reported in older adults [62], and recent component-level work supports age-related anterior redistribution [63]. Importantly, our sensor-level analysis cannot determine whether a single P3 generator shifted anteriorly or whether the pattern reflects changes in the relative contributions of overlapping anterior and posterior activities. We therefore interpret the present findings as an age-related difference in the scalp distribution of late positive activity, not as direct evidence of frontal compensation or generator displacement.

In the beta-band analyses, older adults showed higher prestimulus beta power across the frontal, central, and posterior-parietal ROIs, indicating that age-group differences in beta activity were already present before cue onset. This pattern is consistent with reports of increased resting or spontaneous sensorimotor beta activity in healthy aging [26,27,28]. Because the prestimulus interval was embedded within the task, however, it should be interpreted as the starting level of beta activity for the present task rather than as an independent measure of resting-state activity [64].

Age-group differences were also evident in the fixed 100–500 ms window. Older adults showed a larger reduction in beta power relative to the matched prestimulus reference, while absolute task-period beta also differed between groups after matched prestimulus absolute power was controlled. The age-group difference was therefore present in both the baseline-referenced and baseline-adjusted measures. These measures are related but not interchangeable: when the starting level is higher, a larger relative reduction can coexist with elevated absolute beta during the task [26]. Within the ERD/ERS framework, beta desynchronization has been linked broadly to a shift away from the prevailing sensorimotor state during movement preparation and execution [21,22]. Neither fixed-window measure, however, showed a corrected main effect of coordination mode or an Age Group × Coordination Mode interaction. The standardized early window therefore showed an age-group difference in task-period beta activity but provided no corrected evidence that this difference varied between in-phase and anti-phase coordination.

In contrast to the fixed-window endpoints, cumulative ERD burden showed corrected effects of age group and coordination mode, as well as their interaction, with the largest values in older adults during the anti-phase condition. This pattern accords broadly with reports that beta dynamics vary with bimanual coordination complexity, although age effects have not been uniform across studies [23,24]. The endpoints nevertheless answer different questions: fixed-window power summarizes mean activity during a common early interval, whereas burden integrates the magnitude and temporal extent of below-baseline activity across each detected ERD episode. Their divergent results neither make burden a superior measure nor reveal whether the interaction arose from deeper, longer, or differently timed desynchronization; they show only that coordination-related variation was present in cumulative episode-level activity. Burden should therefore be treated as an exploratory, study-specific cumulative index, and the nonsignificant Coordination Mode × ROI and three-way interactions provide no basis for localizing the effect to one scalp region.

The exploratory logRT and logIES analyses provided no FDR-corrected evidence of brain–behavior associations. These findings do not establish successful compensation, neural inefficiency, or dedifferentiation [14,16].

Several limitations constrain the interpretation of these findings. First, the sample was modest and cross-sectional, and the effective sample differed across different analyses. The smaller samples in the burden analyses, particularly the spatial models, further limit precision. Although all older participants met the MMSE eligibility criterion, this screening measure alone cannot rule out mild cognitive impairment. Recent research in Chinese older adults highlights the importance of considering age and educational background when interpreting MMSE performance [65,66]. The absence of completed education-year records limited assessment of education-related confounding. In addition, practice exposure was not systematically documented, limiting assessment of its potential influence on age-group differences. Secondly, scalp-level ROI analyses provide limited anatomical specificity and cannot identify cortical generators without explicit source modeling [67]. Thirdly, because the P3 (300–600 ms) and beta (100–500 ms) windows overlapped with behavioral response times in several conditions, response-related activity cannot be excluded from these measures. This is particularly relevant for faster trials and younger adults. Thus, the present neural differences should be interpreted as task-related activity that may include motor and response-related contributions, rather than as isolated indices of stimulus evaluation or motor preparation. Future studies using response-locked analyses, response-separated windows, or component decomposition are needed to distinguish these contributions. Finally, the anti-phase condition increased both interlimb coordination demands and stimulus–response mapping complexity, so their respective contributions to the condition effect cannot be separated. Independent replication in larger samples, together with longitudinal designs, task manipulations that disentangle these demands, and source-informed or multimodal measures, is needed to establish the reproducibility and functional significance of the observed age-group differences.

## 5. Conclusions

The present study identified age-related differences in behavioral performance and electrophysiological responses during a discrete bimanual coordination task. Older adults showed poorer performance, particularly during anti-phase coordination. Electrophysiological differences included enhanced early cue-evoked responses, a more anterior pattern of P3 amplitude at the scalp, elevated prestimulus beta power, and task-dependent differences in cumulative beta ERD burden. Examining these complementary measures within a common task extends the characterization of age-related electrophysiological differences during bimanual coordination. However, none of the five neural measures showed a significant association with logRT or logIES after FDR correction. The present analyses therefore do not establish that the measured neural differences explain the behavioral age differences. Nor do they establish the absence of brain–behavior relationships or provide sufficient evidence to distinguish among compensation, dedifferentiation, and neural inefficiency.

## Figures and Tables

**Table 1:** Behavioral performance in the primary analysis cohort (*N* = 42).

Metric	Young In-Phase (*n* = 23)	Young Anti-Phase (*n* = 23)	Older In-Phase (*n* = 19)	Older Anti-Phase (*n* = 19)
Accuracy	0.993 ± 0.021	0.976 ± 0.031	0.973 ± 0.056	0.872 ± 0.130
RT (ms)	543.23 ± 79.21	651.87 ± 120.66	708.86 ± 80.86	938.85 ± 138.65
logRT	6.288 ± 0.139	6.464 ± 0.179	6.558 ± 0.109	6.835 ± 0.144
IES	548.17 ± 87.65	671.28 ± 142.23	729.11 ± 113.36	1130.50 ± 405.95
logIES	6.295 ± 0.150	6.489 ± 0.199	6.582 ± 0.143	6.985 ± 0.290

## Data Availability

The datasets used and analyzed during the current study are available from the corresponding author on reasonable request to protect participant privacy.

## Supplementary Materials

The following supporting information can be downloaded at https://www.mdpi.com/article/10.3390/brainsci16101075/s1: Table S1: Behavioral performance and inferential results in the full recruited cohort (N = 45); Table S2: Full statistical results for the three retained time–frequency endpoints; Table S3: Co-primary brain–behavior models for logIES and logRT.
