# Supplementary material for: Age-Related Electrophysiological Reorganization During In-Phase and Anti-Phase Bimanual Coordination
Source: Brain Sci. 2026 Oct 7;16(10):1075. doi: 10.3390/brainsci16101075 (PMC13702194; doi:10.3390/brainsci16101075)
Supplement: Supplementary file 1 [file brainsci-16-01075-s001.zip › brainsci-4501469-supplementary.pdf]

Supplementary Materials

Supplementary Tables S1–S3

Table S1. Behavioral performance and inferential results in the full recruited cohort (N = 45).

Panel A. Descriptive statistics

Metric	Younger in-phase (n = 23)	Younger anti-phase (n = 23)	Older in-phase (n = 22)	Older anti-phase (n = 22)
Accuracy	0.993 ± 0.021	0.976 ± 0.031	0.973 ± 0.056	0.828 ± 0.184
RT (ms)	543.23 ± 79.21	651.87 ± 120.66	718.05 ± 95.12	962.28 ± 154.12
logRT [ln(ms)]	6.288 ± 0.139	6.464 ± 0.179	6.569 ± 0.124	6.857 ± 0.157
IES	548.17 ± 87.65	671.28 ± 142.23	743.22 ± 132.40	1309.79 ± 755.31
logIES	6.295 ± 0.150	6.489 ± 0.199	6.598 ± 0.161	7.081 ± 0.405

Panel B. Omnibus mixed-design analyses

Metric	Effect	df	F	p	η^2G
Accuracy	Age Group	1, 43	11.84	.001	0.163
Accuracy	Coordination Mode	1, 43	27.04	< .001	0.156
Accuracy	Age Group × Coordination Mode	1, 43	16.72	< .001	0.102
logRT [ln(ms)]	Age Group	1, 43	61.95	< .001	0.565
logRT [ln(ms)]	Coordination Mode	1, 43	266.92	< .001	0.381
logRT [ln(ms)]	Age Group × Coordination Mode	1, 43	15.62	< .001	0.035
IES	Age Group	1, 43	19.80	< .001	0.232
IES	Coordination Mode	1, 43	25.67	< .001	0.171
IES	Age Group × Coordination Mode	1, 43	10.61	.002	0.079
logIES	Age Group	1, 43	44.13	< .001	0.456
logIES	Coordination Mode	1, 43	113.25	< .001	0.325
logIES	Age Group × Coordination Mode	1, 43	20.61	< .001	0.081

Panel C. Holm-adjusted simple contrasts following significant interactions

Metric	Contrast	Estimate	SE	df	t	pHolm
Accuracy	Anti-phase: older − younger	-0.148	0.039	43	-3.79	< .001
Accuracy	In-phase: older − younger	-0.020	0.013	43	-1.56	.125
Accuracy	Older: anti-phase − in-phase	-0.145	0.022	43	-6.50	< .001
Accuracy	Younger: anti-phase − in-phase	-0.017	0.022	43	-0.79	.431
logRT [ln(ms)]	Anti-phase: older − younger	0.393	0.050	43	7.82	< .001
logRT [ln(ms)]	In-phase: older − younger	0.281	0.039	43	7.15	< .001
logRT [ln(ms)]	Older: anti-phase − in-phase	0.289	0.020	43	14.19	< .001

Metric	Contrast	Estimate	SE	df	t	pHolm
logRT [ln(ms)]	Younger: anti-phase − in-phase	0.176	0.020	43	8.86	< .001
IES	Anti-phase: older − younger	638.51	160.31	43	3.98	< .001
IES	In-phase: older − younger	195.05	33.33	43	5.85	< .001
IES	Older: anti-phase − in-phase	566.56	97.32	43	5.82	< .001
IES	Younger: anti-phase − in-phase	123.11	95.19	43	1.29	.203
logIES	Anti-phase: older − younger	0.591	0.094	43	6.26	< .001
logIES	In-phase: older − younger	0.302	0.046	43	6.52	< .001
logIES	Older: anti-phase − in-phase	0.483	0.045	43	10.62	< .001
logIES	Younger: anti-phase − in-phase	0.194	0.044	43	4.36	< .001

Note. Values in Panel A are mean ± SD. Raw RT is retained on the original millisecond scale for interpretability; logRT is the natural logarithm of each participant's condition-level mean completion RT and is the formal RT analysis; raw RT is descriptive only. Raw IES omnibus results are retained as an untransformed comparison analysis. Panel C p values are Holm-adjusted within the corresponding simple-contrast families. IES, inverse efficiency score; η^2_G, generalized eta squared.

Table S2. Full statistical results for the three retained time–frequency endpoints.

Panel A. ROI-factor omnibus models

Endpoint	Effect	F	df	p	qBH	N subjects / rows	Corrected support
Cumulative ERD burden	Age Group	54.60	1.00, 41.80	< .001	< .001	41 / 224	Yes
Cumulative ERD burden	Coordination Mode	68.09	1.00, 112.66	< .001	< .001	41 / 224	Yes
Cumulative ERD burden	ROI	26.97	2.00, 79.16	< .001	< .001	41 / 224	Yes
Cumulative ERD burden	Age Group × Coordination Mode	64.35	1.00, 112.66	< .001	< .001	41 / 224	Yes
Cumulative ERD burden	Age Group × ROI	10.30	2.00, 79.16	< .001	< .001	41 / 224	Yes
Cumulative ERD burden	Coordination Mode × ROI	2.67	2.00, 113.05	.073	.128	41 / 224	No
Cumulative ERD burden	Age Group × Coordination Mode × ROI	2.16	2.00, 113.05	.120	.148	41 / 224	No
Relative beta power, 100–500 ms	Age Group	30.72	1.00, 42.00	< .001	< .001	42 / 252	Yes
Relative beta power, 100–500 ms	Coordination Mode	1.86	1.00, 126.00	.175	.204	42 / 252	No
Relative beta power, 100–500 ms	ROI	30.95	2.00, 84.00	< .001	< .001	42 / 252	Yes
Relative beta power, 100–500 ms	Age Group × Coordination Mode	2.45	1.00, 126.00	.120	.148	42 / 252	No
Relative beta power, 100–500 ms	Age Group × ROI	3.49	2.00, 84.00	.035	.074	42 / 252	No
Relative beta power, 100–500 ms	Coordination Mode × ROI	0.54	2.00, 126.00	.585	.585	42 / 252	No
Relative beta power, 100–500 ms	Age Group × Coordination Mode × ROI	2.66	2.00, 126.00	.074	.128	42 / 252	No
Absolute beta power, 100–500 ms (baseline-adjusted)	Age Group	7.99	1.00, 49.05	.007	.016	42 / 252	Yes
Absolute beta power, 100–500 ms (baseline-adjusted)	Coordination Mode	2.46	1.00, 119.90	.120	.148	42 / 252	No
Absolute beta power, 100–500 ms (baseline-adjusted)	ROI	487.37	2.00, 81.97	< .001	< .001	42 / 252	Yes
Absolute beta power, 100–500 ms (baseline-adjusted)	Age Group × Coordination Mode	1.36	1.00, 121.31	.246	.272	42 / 252	No
Absolute beta power, 100–500 ms (baseline-adjusted)	Age Group × ROI	2.61	2.00, 86.65	.080	.128	42 / 252	No
Absolute beta power, 100–500 ms (baseline-adjusted)	Coordination Mode × ROI	0.60	2.00, 119.87	.552	.580	42 / 252	No
Absolute beta power, 100–500 ms (baseline-adjusted)	Age Group × Coordination Mode × ROI	2.19	2.00, 120.46	.117	.148	42 / 252	No

Panel B. ROI-gate decisions

Endpoint	ROI gate	ROI q	Age × ROI q	Coordination × ROI q	Three-way q	Coordination ROI-specific claim
Cumulative ERD burden	Open	< .001	< .001	.128	.148	No
Relative beta power, 100–500 ms	Open	< .001	.074	.585	.128	No
Absolute beta power, 100–500 ms (baseline-adjusted)	Open	< .001	.128	.580	.148	No

Panel C. Conditional ROI-separated models

Endpoint	ROI	Effect	F	df	p	qBH	N subjects / rows	Baseline covariate	Corrected support
Cumulative ERD burden	Frontal	Age Group	17.42	1.00, 36.85	< .001	< .001	36 / 69	No	Yes
Cumulative ERD burden	Frontal	Coordination Mode	6.65	1.00, 34.91	.014	.015	36 / 69	No	Yes
Cumulative ERD burden	Frontal	Age Group × Coordination Mode	6.57	1.00, 34.91	.015	.015	36 / 69	No	Yes
Cumulative ERD burden	Central	Age Group	54.60	1.00, 41.34	< .001	< .001	41 / 78	No	Yes
Cumulative ERD burden	Central	Coordination Mode	41.92	1.00, 38.12	< .001	< .001	41 / 78	No	Yes
Cumulative ERD burden	Central	Age Group × Coordination Mode	41.55	1.00, 38.12	< .001	< .001	41 / 78	No	Yes
Cumulative ERD burden	Posterior-parietal	Age Group	48.45	1.00, 40.40	< .001	< .001	40 / 77	No	Yes
Cumulative ERD burden	Posterior-parietal	Coordination Mode	34.00	1.00, 38.26	< .001	< .001	40 / 77	No	Yes
Cumulative ERD burden	Posterior-parietal	Age Group × Coordination Mode	26.76	1.00, 38.26	< .001	< .001	40 / 77	No	Yes
Relative beta power, 100–500 ms	Frontal	Age Group	21.75	1.00, 42.00	< .001	< .001	42 / 84	No	Yes
Relative beta power, 100–500 ms	Frontal	Coordination Mode	0.03	1.00, 42.00	.855	.855	42 / 84	No	No
Relative beta power, 100–500 ms	Frontal	Age Group × Coordination Mode	0.33	1.00, 42.00	.570	.641	42 / 84	No	No
Relative beta power, 100–500 ms	Central	Age Group	24.43	1.00, 42.00	< .001	< .001	42 / 84	No	Yes
Relative beta power, 100–500 ms	Central	Coordination Mode	0.42	1.00, 42.00	.520	.641	42 / 84	No	No
Relative beta power, 100–500 ms	Central	Age Group × Coordination Mode	9.74	1.00, 42.00	.003	.007	42 / 84	No	Yes
Relative beta power, 100–500 ms	Posterior-parietal	Age Group	31.80	1.00, 42.00	< .001	< .001	42 / 84	No	Yes
Relative beta power, 100–500 ms	Posterior-parietal	Coordination Mode	2.52	1.00, 42.00	.120	.216	42 / 84	No	No
Relative beta power, 100–500 ms	Posterior-parietal	Age Group × Coordination Mode	0.55	1.00, 42.00	.463	.641	42 / 84	No	No
Absolute beta power, 100–500 ms (baseline-adjusted)	Frontal	Age Group	6.83	1.00, 41.48	.012	.037	42 / 84	Yes	Yes

Endpoint	ROI	Effect	F	df	p	qBH	N subjects / rows	Baseline covariate	Corrected support
Absolute beta power, 100–500 ms (baseline-adjusted)	Frontal	Coordination Mode	0.05	1.00, 40.33	.823	.823	42 / 84	Yes	No
Absolute beta power, 100–500 ms (baseline-adjusted)	Frontal	Age Group × Coordination Mode	0.43	1.00, 40.34	.515	.628	42 / 84	Yes	No
Absolute beta power, 100–500 ms (baseline-adjusted)	Central	Age Group	1.65	1.00, 44.10	.206	.371	42 / 84	Yes	No
Absolute beta power, 100–500 ms (baseline-adjusted)	Central	Coordination Mode	0.72	1.00, 41.18	.401	.601	42 / 84	Yes	No
Absolute beta power, 100–500 ms (baseline-adjusted)	Central	Age Group × Coordination Mode	6.95	1.00, 42.77	.012	.037	42 / 84	Yes	Yes
Absolute beta power, 100–500 ms (baseline-adjusted)	Posterior-parietal	Age Group	15.69	1.00, 41.77	< .001	.003	42 / 84	Yes	Yes
Absolute beta power, 100–500 ms (baseline-adjusted)	Posterior-parietal	Coordination Mode	2.92	1.00, 41.14	.095	.214	42 / 84	Yes	No
Absolute beta power, 100–500 ms (baseline-adjusted)	Posterior-parietal	Age Group × Coordination Mode	0.35	1.00, 41.34	.558	.628	42 / 84	Yes	No

Panel D. Parent-gated coordination simple effects

Endpoint	ROI	Age group	Contrast	Estimate	SE	t (df)	p	qBH	Parent interaction q	N subjects / rows	Corrected support
Cumulative ERD burden	Frontal	Younger	In-phase − anti-phase	-2.11	210.85	-0.01 (35.7)	.992	.992	.015	36 / 69	No
Cumulative ERD burden	Frontal	Older	In-phase − anti-phase	-719.68	184.00	-3.91 (33.9)	< .001	< .001	.015	36 / 69	Yes
Cumulative ERD burden	Central	Younger	In-phase − anti-phase	-3.22	160.98	-0.02 (38.8)	.984	.992	< .001	41 / 78	No
Cumulative ERD burden	Central	Older	In-phase − anti-phase	-1457.94	158.16	-9.22 (37.5)	< .001	< .001	< .001	41 / 78	Yes
Cumulative ERD burden	Posterior-parietal	Younger	In-phase − anti-phase	-71.19	154.19	-0.46 (39.0)	.647	.970	< .001	40 / 77	No
Cumulative ERD burden	Posterior-parietal	Older	In-phase − anti-phase	-1190.13	151.70	-7.85 (37.5)	< .001	< .001	< .001	40 / 77	Yes
Relative beta power, 100–500 ms	Central	Younger	In-phase − anti-phase	-0.124	0.067	-1.84 (42.0)	.073	.220	.007	42 / 84	No
Relative beta power, 100–500 ms	Central	Older	In-phase − anti-phase	0.189	0.074	2.55 (42.0)	.015	.088	.007	42 / 84	No
Absolute beta power, 100–500 ms (baseline-adjusted)	Central	Younger	In-phase − anti-phase	-0.008	0.006	-1.34 (42.4)	.188	.375	.037	42 / 84	No
Absolute beta power, 100–500 ms (baseline-adjusted)	Central	Older	In-phase − anti-phase	0.016	0.007	2.37 (41.7)	.023	.136	.037	42 / 84	No

Panel E. Absolute-power nuisance-control terms

Model	ROI	Endpoint	Control term	F	df	p	N subjects / rows	Multiplicity role
Full ROI-factor model	All ROIs	Absolute beta power, 100–500 ms (baseline-adjusted)	Prestimulus beta	696.57	1.00, 105.72	< .001	42 / 252	Excluded from endpoint FDR and ROI gate

Model	ROI	Endpoint	Control term	F	df	p	N subjects / rows	Multiplicity role
Full ROI-factor model	All ROIs	Absolute beta power, 100–500 ms (baseline-adjusted)	Prestimulus Beta × ROI	4.37	2.00, 86.97	.016	42 / 252	Excluded from endpoint FDR and ROI gate
ROI-separated model	Frontal	Absolute beta power, 100–500 ms (baseline-adjusted)	Prestimulus beta	431.50	1.00, 44.86	< .001	42 / 84	Excluded from endpoint FDR
ROI-separated model	Central	Absolute beta power, 100–500 ms (baseline-adjusted)	Prestimulus beta	254.80	1.00, 49.14	< .001	42 / 84	Excluded from endpoint FDR
ROI-separated model	Posterior-parietal	Absolute beta power, 100–500 ms (baseline-adjusted)	Prestimulus beta	495.27	1.00, 44.72	< .001	42 / 84	Excluded from endpoint FDR

Note. The 21 experimental-design terms in Panel A formed one revised BH-FDR family. Conditional ROI-separated models were fitted only after the ROI gate opened and were adjusted within each prespecified endpoint-specific follow-up family. Panel D reports coordination contrasts only where the corresponding ROI-separated Age Group × Coordination Mode parent effect survived correction; q values were calculated across the six age-by-ROI contrasts for each endpoint. Absolute beta models included matched prestimulus beta and, in the ROI-factor model, its interaction with ROI; these nuisance-control terms were excluded from endpoint FDR and ROI gating. Burden values without a valid detected episode remained missing and were not replaced with zero. A significant result in one ROI does not establish ROI specificity; such a claim requires a corrected Coordination Mode × ROI or three-way interaction in the full ROI-factor model.

Table S3. Co-primary brain–behavior models for logIES and logRT.

Panel A. Neural-feature coefficients and multiplicity-adjusted inference

Outcome	Neural feature	β per 1 SD	95% CI	t (df)	p	qBH	LRT p	LRT qBH	N subjects / rows
logIES	P3 anteriority	0.053	[-0.006, 0.113]	1.80 (65.5)	.076	.127	.069	.115	42 / 84
logIES	Posterior-parietal P3 50% fractional-area latency	0.060	[0.006, 0.115]	2.22 (67.8)	.030	.074	.032	.080	42 / 84
logIES	Cumulative ERD burden	0.082	[0.010, 0.155]	2.26 (74.1)	.027	.074	.022	.080	41 / 80
logIES	Relative beta power, 100–500 ms	-0.020	[-0.089, 0.048]	-0.59 (61.1)	.556	.556	.557	.557	42 / 84
logIES	Absolute beta power, 100–500 ms	-0.105	[-0.294, 0.084]	-1.11 (62.1)	.270	.337	.263	.328	42 / 84
logRT	P3 anteriority	0.029	[-0.012, 0.070]	1.41 (75.5)	.162	.405	.149	.372	42 / 84
logRT	Posterior-parietal P3 50% fractional-area latency	0.017	[-0.021, 0.055]	0.89 (77.4)	.378	.631	.415	.691	42 / 84
logRT	Cumulative ERD burden	0.050	[0.002, 0.098]	2.05 (74.5)	.043	.217	.036	.182	41 / 80
logRT	Relative beta power, 100–500 ms	0.009	[-0.039, 0.057]	0.37 (70.3)	.713	.892	.704	.881	42 / 84
logRT	Absolute beta power, 100–500 ms	-0.004	[-0.135, 0.127]	-0.06 (70.6)	.955	.955	.946	.946	42 / 84
